# Smartphone Usage Patterns Among Postgraduate Medical Students: A Central India Perspective on Adaptive Learning in Medicine

**DOI:** 10.7759/cureus.49549

**Published:** 2023-11-28

**Authors:** Pragati G Rathod, Sarita K Sharma, Ujwala U Ukey, Prashant Ghunkikar, Mithra Prakash, Ajaya Krishnan P

**Affiliations:** 1 Community Medicine, Government Medical College (GMC), Nagpur, IND; 2 Community Medicine, Bharati Vidyapeeth Medical College, Sangli, IND

**Keywords:** postgraduates, m-learning, medical education, social media, smartphone

## Abstract

Introduction

Technological developments are drawn on a path of continuous inventions. Smartphones have been used in educational activities to access course content, acquire information related to students' performance, and encourage discussion and sharing between students and teachers. Students as learners are the drivers of using new technology for their learning needs, and this is always evolving.

Aim

The aim of the study is to assess the usage of smartphones for academic learning among postgraduate medical students in a teaching institute in Central India.

Materials and methods

This cross-sectional, questionnaire-based study was conducted on 130 postgraduate medical students for a period of four months from 1 January 2022 to 30 April 2022.

Results

The mean age of the study subjects was 28.34 ± 3.22 years with the range being 23 to 44 years. Smartphones had a significant impact on the academic learning of postgraduate medical students. The major impacts of smartphones on academic learning were in the form of improved learning skills (60.91%), timely completion of assignments (40%), increased participation in discussions (34.55%), enhanced academic performance (33.64%), and development of critical and innovative thinking (29.09%).

Conclusion

A significant proportion of medical postgraduates are utilizing smartphones and social media for academic purposes. Although this new technology offers the potential to enhance learning and patient care, it has some inherent problems associated with its use. However, it may go a long way in redefining how we manage information in medicine.

## Introduction

In recent years, smartphones have become an integral part of our daily lives, with exponential growth in their usage throughout the world including India [1]. Notably, they have evolved beyond mere communication devices, now serving as powerful tools for academic advancement, commonly referred to as m-learning. Educational institutions worldwide are increasingly embracing smartphone technology to enable students to access course materials, track their performance, and facilitate interaction and collaboration between peers and educators [2].

Moreover, smartphones have significantly impacted medical care, offering a wide array of applications ranging from medical calculators and reference tools to clinical discussion groups on platforms such as WhatsApp (Meta Platforms, Inc., Cambridge, MA). These applications have become invaluable aids for busy healthcare professionals, allowing for enhanced clinical decision-making and knowledge sharing. However, the proliferation of health-related applications raises concerns about the quality, privacy, and evidence-based nature of these tools.

Smartphones play a pivotal role in medical education, offering multifaceted benefits to students. Studies have shown that a substantial proportion of medical students heavily rely on smartphone applications for accessing course content, acquiring information related to students' performance, and discussing and sharing information between students and teachers [2,3]. A study conducted by Robinson et al. demonstrated that 84% of medical students found smart devices to be valuable complements to their education [4].

Smartphones are becoming increasingly common in both personal and professional spheres [5]. Despite the wealth of international research on smartphone usage among medical students, there remains a significant gap in our understanding of smartphone utilization for academic learning among postgraduate medical students. To address this knowledge gap, we have undertaken a study to assess the extent and nature of smartphone usage in the academic pursuits of postgraduate medical students at a teaching institute located in Central India.

## Materials and methods

Study design, setting, and duration

The present descriptive, cross-sectional study was conducted in a postgraduate institute in Central India. The study spanned a period of four months from 1 January 2022 to 30 April 2022.

Study population

Postgraduate students from preclinical, paraclinical, medicine-allied, and surgery-allied specialties constituted the study population.

Sample size calculation and sampling technique

Based on a previous study conducted by Haseeb et al. in Jammu, assuming a proportion of smartphone usage for academic learning among postgraduate students as 90% and taking absolute precision of 6% and a confidence interval of 95%, the estimated sample size was 96. The study subjects were recruited by convenience sampling method [6].

Data collection

The data collection tool constituted a self-administered questionnaire (Appendices). The questionnaire was developed by the lead researcher, underwent content validity and reliability assessment by an expert panel, and was refined based on pilot testing with interns. The link to the questionnaire was distributed via WhatsApp using a recognized survey website (docs.google.com, Google, Inc., Mountain View, CA). The link was sent to 130 postgraduate students from various specialties, of which 112 responded completely, resulting in a response rate of 86.15%. The questionnaire collected data on the following variables: demographic details, frequency and purposes of smartphone usage for academic learning, specific applications used for academic learning, social media platforms utilized for academic purposes, and perceived advantages of smartphone use in academics.

Data analysis

The data generated through the questionnaire was entered into Microsoft Excel (Microsoft® Corp., Redmond, WA) for analysis. Frequencies and percentages were calculated to summarize the data.

Ethical considerations

Ethical clearance was obtained from the Institutional Ethics Committee at the Government Medical College, Nagpur, as per letter number 1874 dated 16 September 2022. Permission was taken from the institutional head and respective professor and head of various departments at the Government Medical College, Nagpur. Written informed consent was obtained from the study subjects, after providing them with information about the nature and purpose of the study. The participants were assured of confidentiality and anonymity, by excluding personal details such as name, address, and contact number from the questionnaire.

## Results

Sociodemographic characteristics are shown in Table 1. Of the 112 study subjects, 66 (58.9%) were males and 46 (41.1%) were females. Almost 85% of the study subjects were less than 30 years of age with a mean age of 28.34 ± 3.22 years and the range being 23-44 years. As per the year of postgraduation, 29.46%, 34.82%, and 35.71% of students were studying in the first, second, and third years, respectively. Maximum study subjects were from medicine-allied specialties (46, 41.1%), followed by surgery-allied (37, 33%). The majority of the study subjects (74, 66.1%) were of urban origin.

**Table 1:** Sociodemographic characteristics of the study participants

Variable	Number	Frequency
Age group (years)		
≤30	95	84.8
≥30	17	15.2
Gender		
Male	66	58.9
Female	46	41.1
Year of postgraduation		
1st year	33	29.5
2nd year	39	34.8
3rd year	40	35.7
Specialties		
Preclinical	8	7.1
Paraclinical	21	18.8
Medicine-allied	46	41.1
Surgery-allied	37	33.0
Place of origin		
Urban	74	66.1
Rural	38	33.9

**Figure 1:**
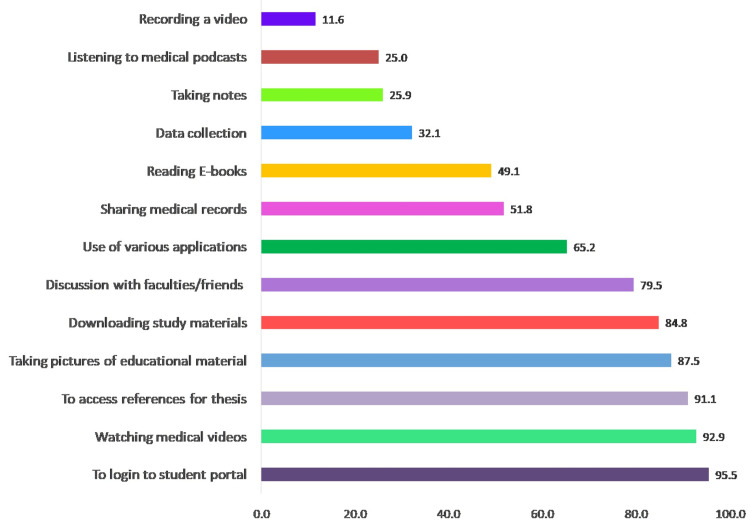
Use of smartphones for academic activities among the study participants

All (112) study participants owned a smartphone and used it for some or other academic reason. The use of smartphones in academic activities refers to their practical application as tools for accessing educational resources and communication. As can be seen from Figure 1, smartphones were mainly used for logging into the student portal (107, 95.5%), watching medical videos (104, 92.9%), accessing references for thesis (102, 91.1%), and downloading study material (95, 84.8%).

**Figure 2:**
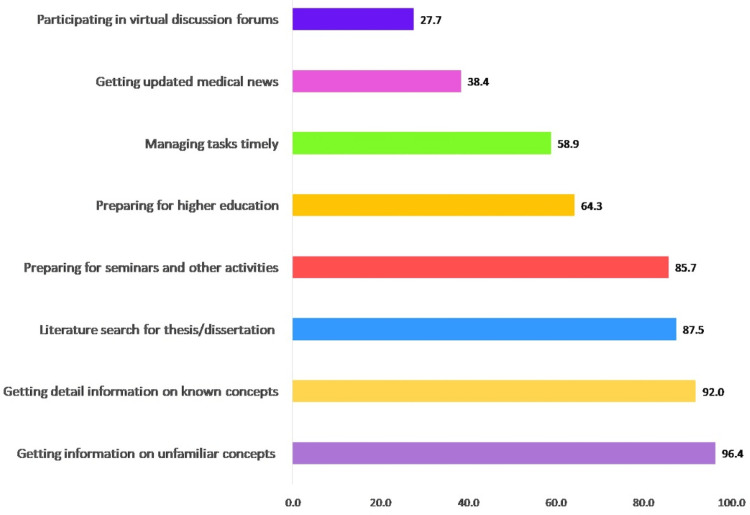
Purpose of using smartphones among the study participants

The purpose of smartphone use in academics encompasses their broader role in facilitating learning, collaboration, and digital engagement within the educational environment. The main purpose of using smartphones by the study subjects was to get information on different concepts, conduct a literature search for thesis and projects, and prepare for seminars and other postgraduate activities as can be seen in Figure 2.

**Figure 3:**
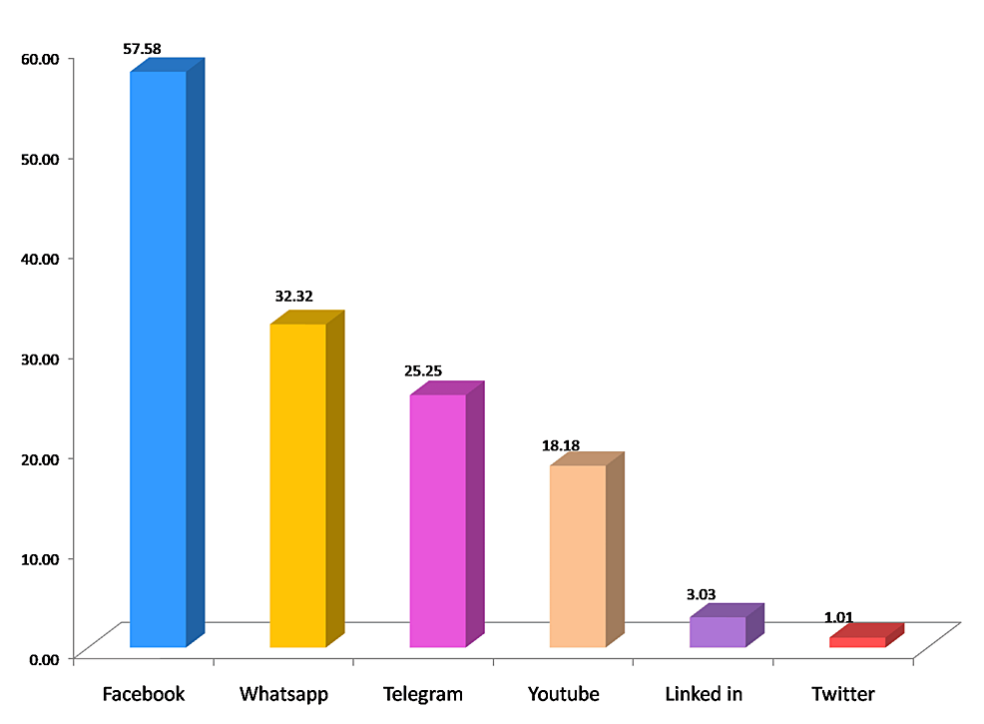
Social media applications used for academic learning

Of the 112 study participants, 99 (88.4%) used social media for academic learning, and the most commonly used social media for this purpose were Facebook, WhatsApp, and Telegram (Figure 3).

**Figure 4:**
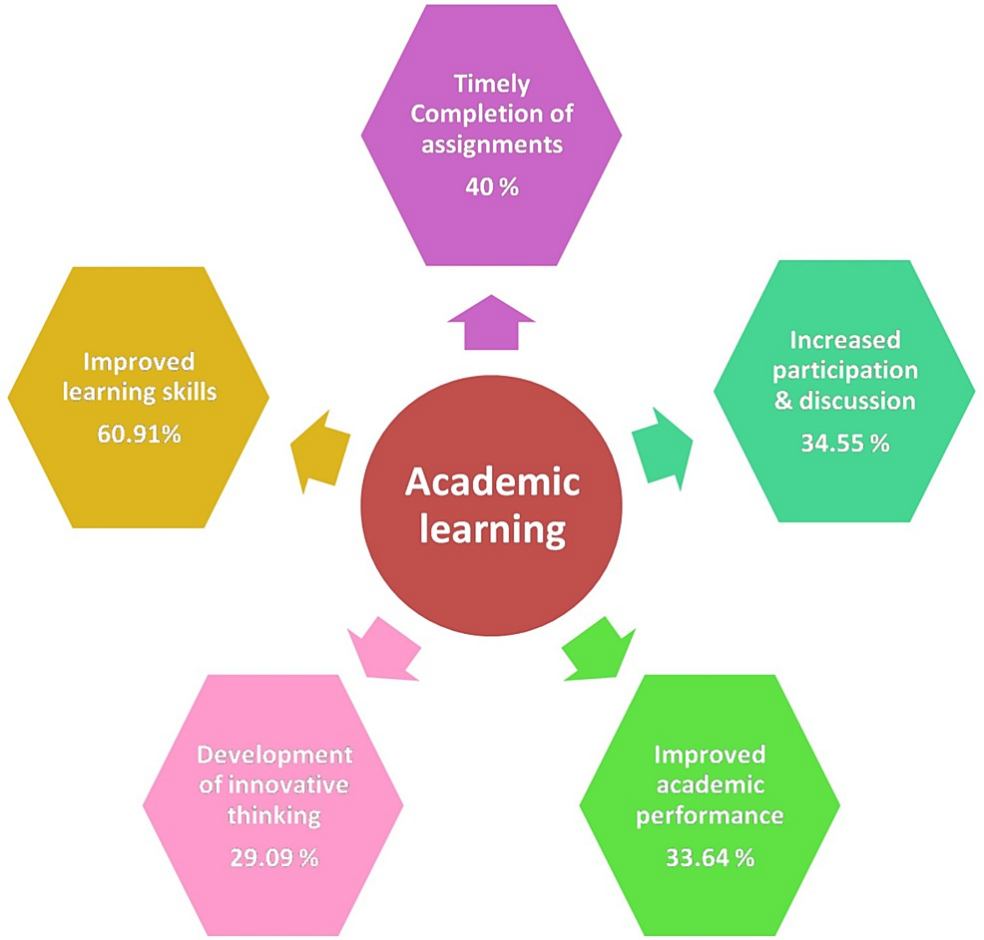
Impact of smartphones on academic learning

With their versatile capabilities and easy accessibility, smartphones have become valuable educational tools that enhance the learning experience in several ways. Smartphones had a significant impact on the academic learning of postgraduate medical students as depicted in Figure 4. The major impacts of smartphones on academic learning were in the form of improved learning skills, the timely completion of assignments, increased participation in discussions, enhanced academic performance, and the development of critical and innovative thinking.

The main advantages of smartphones as described by the study subjects were being helpful in finding updated information, saving time, facilitating learning, and improving searching. Some limitations of smartphones enlisted by the study subjects were the high cost of applications, distraction from studies, battery issues, problems with internet access, etc. (Table 2).

**Table 2:** Advantages and limitations of smartphone usage

Advantages of smartphones	Frequency	Percentage
Help find updated information	77	70
Save time and increase productivity	69	62.73
Facilitate learning	62	56.36
Improve searching and learning skills	62	56.36
Limitations of the use of smartphones		
High cost of applications	79	70.5
Distraction from studies	52	46.4
Small screen size	49	43.8
Issues with battery charging	38	33.9
Small keyboard	32	28.6
Problems with internet access	25	22.3
High cost of network services	18	16.1

## Discussion

In the contemporary era, where smartphones and modern information technology have fundamentally transformed the way we access and disseminate information, our study aimed to evaluate the utilization of smartphones for academic purposes among postgraduate medical students within a tertiary healthcare institution. Our findings revealed that all study participants were smartphone owners and utilized these devices for various academic pursuits. This trend aligns with prior research conducted in India, where 96% of medical students were reported to own smartphones, with 90% of them possessing the necessary skills for using these devices in the context of medical education [7].

Our study participants predominantly harnessed the potential of smartphones as versatile tools for educational purposes and communication. They commonly utilized their smartphones to log into their student portals, watch medical videos, access references for their theses, and capture images of academic materials. Additionally, smartphones played a substantial role in facilitating learning and digital communication. The participants frequently used their smartphones to gather information on both unfamiliar and familiar concepts, enhancing their educational experience. Moreover, they employed these devices for dissertation-related literature searches and preparations for seminars and postgraduate activities. This multifaceted use of smartphones underscores their significance as invaluable companions in the academic pursuits of postgraduate medical students.

A study conducted in Saudi Arabia by Jamal et al. found that, besides communication, residents primarily used smartphones for accessing drug references (82.2%), medical references (79.2%), medical calculations (60.4%), medical photography, and literature searches [8]. In a systematic review by Sterling et al., it was revealed that residents attempted to enhance their educational experience by leveraging social media, with platforms such as Twitter, podcasts, and blogs being the most frequently used. Less common platforms included wikis (15.4%), Skype (7.7%), and YouTube (7.7%) [9]. In our study, we observed that 88.4% of the participants accessed social media for academic learning through smartphones, with Facebook being the most popular choice (57.58%). Other platforms included WhatsApp (32.32%), Telegram (25.25%), and YouTube (18.18%). These variations in platform preferences could be attributed to contextual differences.

Significantly, a considerable number of our study participants expressed the view that smartphones played a constructive role in their academic endeavors. A notable percentage believed that these devices had a positive influence on their learning skills, assisting them in achieving better results. Many also found that smartphones contributed to the timely completion of assignments, while a substantial portion reported enhanced academic performance. This perception aligns with the research conducted by Subhash and Bapurao, who similarly found that a majority of undergraduate medical students considered mobile technology to be a viable and advantageous asset in the realm of medical education [10].

However, it is important to acknowledge that smartphones are not without limitations. The high cost of various services, limited screen size, issues with battery life, and network access problems emerged as significant drawbacks in our study. These findings are consistent with those of other similar studies [11-15].

The study has some intrinsic limitations of a typical cross-sectional study such as providing only a snapshot at a particular point in time, thus making it difficult to establish a causal relationship. Also, the convenience of using an online platform for data collection may introduce selection bias because individuals who are already comfortable with and have access to these technologies are the ones who are more likely to respond. This limits the generalizability of the study findings to a broader, potentially more diverse population of medical postgraduates.

There is a need for carrying out more studies with larger sample sizes and different study designs to comprehensively understand how this technology can positively transform learning outcomes and interprofessional relationships. Integrating qualitative methods to gather inputs from students about their smartphone practices could provide in-depth insight for integrating these digital tools alongside or in tandem with traditional approaches to education.

## Conclusions

A significant proportion of medical postgraduates are utilizing smartphones and social media for academic purposes. This emerging technology holds the potential to bolster educational experiences and improve patient care. The impact of smartphones on academic learning was significant, leading to improvements in learning skills, timely assignment completion, increased participation in discussions, enhanced academic performance, and the development of critical and innovative thinking. These findings accentuate the valuable role of smartphones in enhancing the academic experiences of postgraduate medical students, emphasizing their potential for improving educational outcomes.

Despite introducing potential challenges, smartphone usage has the capacity to redefine information management practices in the medical field. Leadership within medical schools and healthcare organizations must proactively guide developments in this rapidly evolving landscape. Initiating a constructive dialogue with users is essential to gain insights into their specific needs and preferences, with the overarching goal of maximizing the advantages of this powerful technology while being vigilant about unintended consequences. The study's results emphasize the necessity of integrating smartphone-based resources and tools in medical education to further enhance the learning process and ultimately benefit postgraduate medical students.

## Appendices

Questionnaire

Smartphone Usage Patterns Among Postgraduate Medical Students: A Central India Perspective on Adaptive Learning in Medicine

Designation:                              Discipline:                 Age:                          Gender:                              Residence: Rural/Urban

1. Do you use a smartphone? Yes/No

1a. If yes, do you use your mobile device or phone for academic purposes?

2. List the academic activities for which you use your smartphone:

a. Data collection

b. Discussion with faculties/friends 

c. Downloading study materials

d. Listening to medical podcasts

e. Reading E-books

f. Recording a video

g. Sharing medical records

h. Taking notes

i. To access references for thesis

j. Use of various applications

k. Watching medical videos

l. Others (specify): _________________________________________

3. Do you use any applications related to your subject?

3a. If yes, enlist the names and uses:

___________________________

___________________________

___________________________

4. List the purpose for which you use your smartphone:

a. Getting detailed information on known concepts

b. Getting information on unfamiliar concepts 

c. Literature search for thesis/dissertation 

d. Managing tasks timely

e. Participating in virtual discussion forums

f. Preparing for higher education

g. Preparing for seminars and other activities

h. Others (specify): _________________________________________

5. Which social media and networking sites do you use for academic learning?

___________________________

___________________________

___________________________

6. How do smart smartphones impact academic learning?

a. Improved my learning skills

b. Helped to complete my assignments on time

c. Motivated for active participation and discussion

d. Cultivated innovative thinking

e. Improved academic performance

f. Others (specify): _________________________________________

7. What are the advantages of smartphones in academic learning?

a. Facilitates learning

b. Helps to find updated information

c. Increases searching and learning skills

d. Saves time and increases productivity

8. What are the limitations of using smartphones in academic learning?

a. Distraction from studies

b. High cost of applications

c. High cost of network services

d. Issues with battery charging

e. Problems with internet access

f. Small keyboard

g. Small screen size
